# The role of the immune system in idiopathic nephrotic syndrome

**DOI:** 10.1186/s40348-021-00128-6

**Published:** 2021-11-18

**Authors:** Agnes Hackl, Seif El Din Abo Zed, Paul Diefenhardt, Julia Binz-Lotter, Rasmus Ehren, Lutz Thorsten Weber

**Affiliations:** 1 Department of Pediatrics, University of Cologne, Faculty of Medicine and University Hospital Cologne, Cologne, Germany; 2 Department of Internal Medicine II and Center for Molecular Medicine Cologne, University of Cologne, Faculty of Medicine and University Hospital Cologne, Cologne, Germany

**Keywords:** Idiopathic nephrotic syndrome, T-cell dysregulation, Regulatory T-cells, Th17-cells, Podocytes, Antigen-presenting cell

## Abstract

Idiopathic nephrotic syndrome (INS) in children is characterized by massive proteinuria and hypoalbuminemia and usually responds well to steroids. However, relapses are frequent, which can require multi-drug therapy with deleterious long-term side effects. In the last decades, different hypotheses on molecular mechanisms underlying INS have been proposed and several lines of evidences strongly indicate a crucial role of the immune system in the pathogenesis of non-genetic INS. INS is traditionally considered a T-cell-mediated disorder triggered by a circulating factor, which causes the impairment of the glomerular filtration barrier and subsequent proteinuria. Additionally, the imbalance between Th17/Tregs as well as Th2/Th1 has been implicated in the pathomechanism of INS. Interestingly, B-cells have gained attention, since rituximab, an anti-CD20 antibody demonstrated a good therapeutic response in the treatment of INS. Finally, recent findings indicate that even podocytes can act as antigen-presenting cells under inflammatory stimuli and play a direct role in activating cellular pathways that cause proteinuria. Even though our knowledge on the underlying mechanisms of INS is still incomplete, it became clear that instead of a traditionally implicated cell subset or one particular molecule as a causative factor for INS, a multi-step control system including soluble factors, immune cells, and podocytes is necessary to prevent the occurrence of INS. This present review aims to provide an overview of the current knowledge on this topic, since advances in our understanding of the immunopathogenesis of INS may help drive new tailored therapeutic approaches forward.

## Introduction

Idiopathic nephrotic syndrome (INS) is the most frequent glomerular disease in childhood and is caused by damage to podocytes, resulting in foot process effacement that leads to alterations to the selectivity of the glomerular filtration barrier [1]. It is characterized by episodes of severe proteinuria and hypoalbuminemia often associated with dyslipidemia and edema [1]. Loss of serum proteins leads to a hypercoagulable state, a higher rate of infectious diseases, and fluid balance dysregulation. It affects two to ten children per 100,000 per year, with a cumulative prevalence of 16 per 100,000 children [1]. Electron microscopy examination of renal biopsies reveals diffuse foot process effacement, while renal histology shows either minimal podocyte changes without deposition of antibodies termed minimal change disease (MCD) or focal and segmental glomerulosclerosis (FSGS). Evolution from MCD to FSGS is also possible over time [2]. Although most patients respond favorably to steroids, the relapse rate is as high as 80%, and a long-term combination of steroids and/or alternative immunosuppressive agents are often required to maintain remission [3–5].

Several lines of evidence strongly indicate a role of the immune system in the pathogenesis of non-genetic INS. Under these are the effectiveness of immunosuppressive therapies, a frequent remission after measles infection, which leads to cell-mediated immunosuppression, and the association of MCD with T-cell lymphomas [6]. In addition, serum from patients with post-transplant relapse of INS as well as supernatants from T-cell hybridomas from individuals with MCD can induce proteinuria in rats [6, 7].

Soluble factors, different immune cells, recently even immunologic properties of podocytes are potentially implicated in the pathogenesis of the disease, but the precise role of the immune system in INS has not yet been completely elucidated. This review aims to provide an overview of current knowledge on the immune system influencing the course of INS and its response to initial steroid treatment, since advances in our understanding of the pathogenesis of INS may help drive new tailored therapeutic approaches forward.

### Trigger events

Whereas INS usually arises in healthy children, disease onset and relapses are often associated with intercurrent infections and other immunological triggers. Vaccination and atopy have been described in patients with relapse, suggesting that immune activation is involved in INS exacerbation [8–11]. Upper airway infections have been reported as the most frequent infections causing nephrotic relapse [12–14], and interestingly its likelihood decreases with corticosteroid treatment during intercurrent infections [15, 16]. Also, COVID-19 has been reported as a trigger for recurrence of INS [17]. The immune responses prompted by the infection can lead to relapse by generating pathogen and danger-associated molecular patterns that stimulate Toll-like receptors (TLR) and the complement system. These innate immune reactions activate immune cells to release inflammatory mediators and initiate adaptive, antigen-specific immune responses and induce CD80 expression on podocytes that may directly lead to podocyte injury and foot process effacement (see below).

### T-cells and their cytokines

Shalhoub et al. hypothesized that INS represents the renal manifestation of a systemic T-cell dysregulation (deficient suppression) resulting in the production of a circulating mediator, which modifies podocyte structure, leads to foot-process effacement and results in so-called lipoid nephrosis [6, 18, 19]. Compelling evidence for this disease mechanism stems from numerous clinical observations of disease recurrence immediately post-transplant [20, 21] and of trans-placental transmission of the “permeability factor” leading to neonatal transient proteinuria [22]. This hypothesis is further supported by the absence of immune complexes in glomeruli, a frequent remission after measles infection, as well as the association of MCD with T-cell lymphomas [6]. Finally, the findings that injection of supernatants from T-cell hybridomas from patients with MCD orserum from patients with post-transplant relapse of INS can induce proteinuria in rats [7, 23], strengthen the relationship between dysregulated T-lymphocytes and the development of INS.

Different subsets of T-cells have been implicated in the pathogenesis of INS in the last decades. The Immune dysregulation Polyendocrinopathy, Enteropathy, and X-linked (IPEX) syndrome with concomitant nephrotic syndrome provides strong evidence for the crucial role of regulatory T-cells (Tregs) in INS. IPEX syndrome is a rare disorder of the immune regulatory system caused by mutations of forkhead box P3 (FoxP3), which is a transcription factor responsible for the generation and maturation of Tregs (CD4+ CD25+ FoxP3+) [24]. Treg number in physiological states is low, but they can be rapidly generated from immature CD4+ T-cells, that expand in response to stimulation. Several studies in experimental models support the association between low Tregs during a trigger event and proteinuria [25, 26]. For instance, Wang et al. showed that depletion of CD4+ T-cells in BALB/c mice leads to the aggravation of adriamycin-induced nephropathy, while reconstitution with FoxP3-expressing CD4+ CD25+ T-cells ameliorates this disease [27, 28]. In the same model, inducing the expansion of Tregs by administering IL-2/IL-2Ab complexes improved renal function, histological findings, and reduced inflammation [29]. Another study shows that direct infusion of Tregs into Buffalo/Mna rats, a strain that spontaneously develops glomerulosclerosis, is also associated with reduction of proteinuria and amelioration of histological lesions [30]. In lipopolysaccharide (LPS) nephropathy, which represents a model of transient proteinuria, the Treg level was modulated by the administration of IL-2/anti IL-2 immunocomplexes, resulting in a transient protective effect on proteinuria [31]. Human findings support this implication: Benz et al. investigated renal biopsies from 38 pediatric patients and found that the number of FoxP3+ T-cells was significantly lower in MCG and FSGS patients compared to controls while also being exclusively located in the tubulointerstitium, but not in glomeruli [32]. FACS analysis of peripheral blood mononuclear cells from INS patients revealed decreased levels of Tregs [33, 34]. In line with that, the Treg-related regulatory cytokine IL-10 was significantly decreased in lipoid nephrosis [35]. Furthermore, Araya et al. observed that the percentage of Tregs was similar in healthy controls and patients with INS. However, in co-culture of Tregs and effector T-cells (Teffs) from patients with relapse, the concentration of the regulatory cytokine IL-10 was reduced indicating a deficient suppressor function [36]. Interestingly, Shimada et al. showed that Tregs have the potential to turn off CD80 expression on podocytes once it is induced (see below). Subsequently, Tregs dysfunction could make transient proteinuria persistent, leading to podocyte injury [37]. Finally, Treg dysregulation can amplify the neutrophil-induced oxidative burst triggered by an infection, potentially leading to INS relapse [25]. Finally, a clinical relevant point is that a higher ratio of Tregs to Teffs favors steroid sensitivity (steroid sensitive NS [SSNS]) and a reverse ratio points to steroid resistant NS (SRNS) [38, 39].

Another important cell type, Th17cells also derive from the naïve CD4+ progenitor cells, as do Tregs. These two subsets have antagonist effects: a high Th17/Tregs ratio maintains inflammation, while low ratios lead to suppression of inflammatio n[40]. Liu et al. demonstrated that induction of IL-17 released by Th17 cells plays a key role in adriamycin-induced nephrosis most likely through downregulation of phospho-nephrin and Bcl-2 level via overproduction of c-mip [41]. In line with that, May et al. treated human podocytes with supernatants from Th17cells of healthy controls as well as with serum of patients with INS and found significant stimulation of Janus kinases and mitogen-activated protein kinase pathways and an increase in motility of podocytes [42]. Human studies confirm these data: the frequency of mRNA transcripts of Th17 cell-related factors, such as IL-17 or retinoid orphannuclear receptor, was increased in the blood of patients with INS, while it was associated with a decreased number of Tregs [33]. IL-17 expression in respective kidney biopsies revealed higher expression in INS cases compared to healthy controls. Additionally, positive immunostaining for IL-17 was detected in the glomerular compartment [33]. These results were correlated with higher frequencies of circulating Th17 cells and mRNA levels of Th17cell-associated factors in children with INS [43]. In line, Ye et al. showed an excessive increase of Th17 cells by analyzing the peripheral blood of INS patients [34]. Given that Th17 cells have recently been reported to be resistant to glucocorticoid treatment, and glucocorticoid resistance remains a major challenge in the management of INS [42], it can be of importance to delineate the individual immune profile, including the ratio between Th17 and Treg cells, in order to find the most appropriate therapeutic approach in SRNS.

Among Teffs, the Th2 subset was traditionally indicated as a major player in the pathogenesis of INS, because MCD is often associated with atopy and allergy, which in turn are caused by Th2 immunologic responses [44–49]. The increased serum immunoglobulin(Ig) E level and preservation of IgG4 observed in MCD are also characteristic of a Th2 response [50–52]. Furthermore, Buffalo/Mna rats, which develop MCD spontaneously and FSGS over time, were characterized by Th2 polarization and presented a predominant increase in IL-4 and IL-13 levels, preceding the development of nephrotic syndrome [30, 53]. Indeed, the role of the Th2 subset is also supported by the observation of a specific cytokine profile in patients with MCD [36, 54–56]. One of the strongest candidates is IL-13; studies have identified that increased IL-13 expression by CD3+ T-cells can lead to podocyte injury and induce INS in children or MCD-like phenotype in rats [52, 54, 57–59]. Notably, overexpression of IL-13 caused the downregulation of nephrin and podocin and increased IL-13 induced an upregulation of CD80 (see below). In contrast, IL-9, a cytokine also attributed to Th2 cells, was shown to protect podocytes from excessive damage in adriamycin-induced podocytopathy, underscoring the varying role of Th2 cells [60]. Notably, some observations negate a crucial role of Th2 cells in the pathogenesis of INS [61–63].

Similar to the dichotomy of Tregs/Th17, some findings indicate that the ratio of the Th2/Th1 subsets is much more important in the pathomechanism of INS rather than their absolute count. RNA analysis of rat kidney samples to assess the T-cell infiltrate revealed a cytokine transcript expression profile prompting an involvement of Th2 cells, while a downregulation of Th1 cell cytokines was detected [53]. This shift towards a Th2 phenotype was also observed in children with nephrotic syndrome [64], which was further confirmed by a study analyzing the immune profile in peripheral blood of SSNS patients and SRNS patients, showing an imbalance of Th1 and Th2 [34].

The disproportion between CD4+ and CD8+ T-cells seems to play a relevant role in INS, too. Most patients are found to demonstrate a reduction in CD4+ circulating T-cells and a higher prevalence of CD8+ T-cells during the active phases of disease [55, 64].

### B-cells, their antibodies, and cytokines

Although INS has been traditionally considered to be a T-cell mediated disease [6], recently the view shifted towards a potential role of B-cells in the pathogenesis of INS. A case report described a SDNS patient, who received an anti-CD20 antibody, rituximab (RTX), for severe idiopathic thrombocytopenic purpura and reached not only a normal thrombocyte count but also stable remission of proteinuria [65]. Since then, the number of observations and trials reporting successful treatment of nephrotic patients with RTX has been growing consistently, strongly suggesting an involvement of B-cells in INS pathogenesis. Additionally SSNS patients reportedly have higher B-cell levels at disease onset and during relapse, which only normalize when the patient goes into remission [66]. RTX seems to be particularly effective in steroid- or drug-dependent forms of INS [67, 68] and might even pose as an option for a subset of drug-resistant cases [69]. Taking a look at RTX treatment response can provide insights into particularities of subgroups of INS patients. Ding et al. for example found that patients that had an initial steroid sensitivity and only experienced steroid resistance secondarily, had a higher risk for relapse after kidney transplantation, than patients experiencing steroid resistance from the beginning of their disease. This finding suggests a circulating factor, possibly immune-mediated, as a pathogenic driver [70]. It was also found that roughly two-thirds of patients with secondary steroid resistance reach a complete remission and do not progress any further if treated with RTX, compared to only 26% of patients with initial steroid resistance [71]. Finally, Trautmann et al. were able to show that RTX could be as effective as CNI for SRNS [72]. Taken together, these findings point out the heterogeneity of immunological mechanisms of INS. The effectiveness and safety of RTX for INS are summarized by other reviews [73, 74]. How exactly RTX-mediated B-cell depletion asserts its favourable effect on INS is still unknown. Colucci et al. found the delayed reconstitution of switched memory B-cells after RTX therapy, to be a protective factor against relapse [75]. Others have suggested a direct effect of RTX on the surface of podocytes through an off-target binding to sphyngomyelin-phosphodiesterase-acid-like 3b, thereby exerting a protective effect on the actin cytoskeleton and preventing apoptosis induced by patient sera [76]. However, Kim et al. claim this to be an unspecific finding due to the fixation process of cultured cells, while their study implies local cytokine release by B-cells as the pathogenic effector on podocytes [77].

Previous data had already pointed to a possible role of immunoglobulins as a binding partner to a circulating permeability factor [78]. More recent research suggests the cross-reaction of an antibody against EBNA-1, a protein of the Epstein–Barr virus, with an intracellular podocyte protein as a possible cause of podocyte depletion and subsequent proteinuria [79]. Delville et al. even identified a seven-antibody panel, with the capability to predict the recurrence of FSGS after kidney transplantation at an accuracy of 92%. In this panel, auto-anti-CD40 antibodies were the strongest singular predictor of recurrence, with an accuracy of 76%. If isolated out of patient sera, anti-CD40 antibody showed the potential to induce podocyte damage in cell culture, as well as the ability to amplify damage induced by soluble urokinase-type plasminogen activator receptor (suPAR) through a cooperative effect [80]. Interestingly sCD23, an IgE receptor and B-cell activation marker, was found to be increased during relapse of INS, adding to the assumption of a B-cell dysregulation [81]. Another finding worth mentioning is the significant association of polymorphisms in HLA-DQ1 with SSNS. These polymorphisms could, among other things, lead to a defective antigen presentation, resulting in an abnormal T-cell response [82]. In conclusion, an active role of B-cells in INS pathogenesis gains more and more evidence, challenging the standpoint of INS as a disease only driven by T-cell dysfunction and RTX and other anti-CD20 antibodies pose as promising alternatives to other steroid-sparing agents.

### Mononuclear phagocytes and their cytokines

In Buffalo/Mna rats, which develop INS spontaneously, the frequency of monocyte–macrophage lineage cells and the expression of macrophage-associated factors (tumor necrosis factor-α [TNF-α], IL-12) were found to be higher in the kidney infiltrate compared to healthy rats [53]. Interestingly, this infiltration was already prominent at a non-proteinuric stage before the onset of the disease. The examination of adriamycin-induced nephropathy revealed initial interstitial accumulation of macrophages as well [27, 83, 84] with a subsequent reduction of them during the course of the disease [84]. This study has not detected any glomerular macrophage infiltration. Whether macrophages contribute to early podocyte damage or rather act profibrotic in later stages remains to be elucidated. Corresponding to these animal studies, significantly higher numbers of interstitial CD68+ macrophages were detected in kidney samples from children with INS compared to controls, while the frequency of macrophages was higher in the kidney infiltrate of the FSGS group in comparison to the MCD group [32].

### Circulating permeability factors

In addition to cytokines and immunoglobulins, other circulating factors have been described in the past years, which are only partially of immune origin [18, 85].

uPAR assembles αvβ3 integrin and activates a signaling cascade modifying adhesion to the extracellular matrix and is expressed by several immune cells, but also by endothelial cells and by podocytes [86]. This receptor is functional in maintaining podocyte shape and sieving properties of glomeruli [87] and its soluble form suPAR has been shown to be increased in the plasma of FSGS patients [88] as well as to positively correlate with the degree of podocyte effacement [89]. However, plasma suPAR levels are influenced by renal function and are elevated in other kidney and liver diseases, too [19, 90].

Cardiotrophin-like cytokine factor 1 (CLCF-1), a member of the IL-6 family, is expressed by several tissues and is known to activate B-cells. It was identified in plasma samples of patients with post-transplant recurrence of FSGS [91]. In line with that, CLCF-1 induces albuminuria in mice and increases albumin permeability in isolated rat glomeruli through the activation of the Janus kinases and pathway, which can be reversed by incubation with anti-CLCF-1 antibodies [91].

Hemopexin, an acute phase protein with anti-oxidant function [92], has been shown to reduce the expression of the glomerular glycocalyx and to alter the integrity of the actin cytoskeleton [93]. In line with that, its active form in circulation was found to be highly increased during relapses of MCD [94–96]. Clinically important, proteomics analysis of plasma samples showed that hemopexin can discriminate patients with SSNS versus SRNS pre-treatment [97].

The glycoprotein, angiopoietin-like 4 (Angptl4), inhibits endothelium-bound lipoprotein lipase activity resulting in increased plasma triglyceride levels [98]. Clement at al. reported that glomerular expression of Angptl4 is highly upregulated in serum and podocytes in experimental models of MCD and in human disease [99]. Additionally, podocyte-specific transgenic overexpression of Angptl4 in rats induced nephrotic-range proteinuria, loss of glomerular basement membrane charge, and foot process effacement. It has been proposed that podocytes secrete a hyposialylated form of Angptl4 in MCD, whereas extrarenal organs secrete a sialylated form of Angptl4 in response to an elevated plasma ratio of free fatty acids to albumin. These circulating pools of Angptl4 may reduce proteinuria by interacting with glomerular endothelial β5-integrin [100]. However, progressive accumulation and clustering of Angptl4 in the glomerular basal membrane (GBM) likely activates signals at the podocyte-GBM interface and induces foot-process effacement resulting in proteinuria [101]. Expression of Angptl4 was shown to be decreased upon glucocorticoid administration suggesting a possible role for this protein in SSNS [99], which was confirmed by showing a 16-fold higher Angptl4 level in patients with SSNS relapse vs. in ones with SRNS [102].

Cathepsin-L is an endoprotease responsible for the breakdown of lysosomal proteins. The actin-binding and stabilizing protein synaptopodin is a substrate for cathepsin-L, which thereby has an effect on cytoskeleton organization [103]. Renal cathepsin is overexpressed in both puromycine aminonucleoside nephrosis as well as LPS nephropathy [104] and cathepsin-L knockout mice are protected against LPS nephropathy.

### Podocytes as antigen-presenting cells

A new paradigm for the pathogenesis of proteinuria in nephrotic syndrome has emerged after the discovery by Kestila et al. [105] that mutations in the gene NPHS1, which encodes the podocyte-expressed nephrin, cause congenital NS in humans. Additionally, the development of proteinuria in LPS-injected severe combined immunodeficient (SCID) mice, which are devoid of T- and B-cells, suggests that this mouse model of INS may be independent of T- or B-cells [106]. Based on these data, podocytes have attracted particular attention as a key player in the pathogenesis of INS [107, 108].

**Fig. 1:**
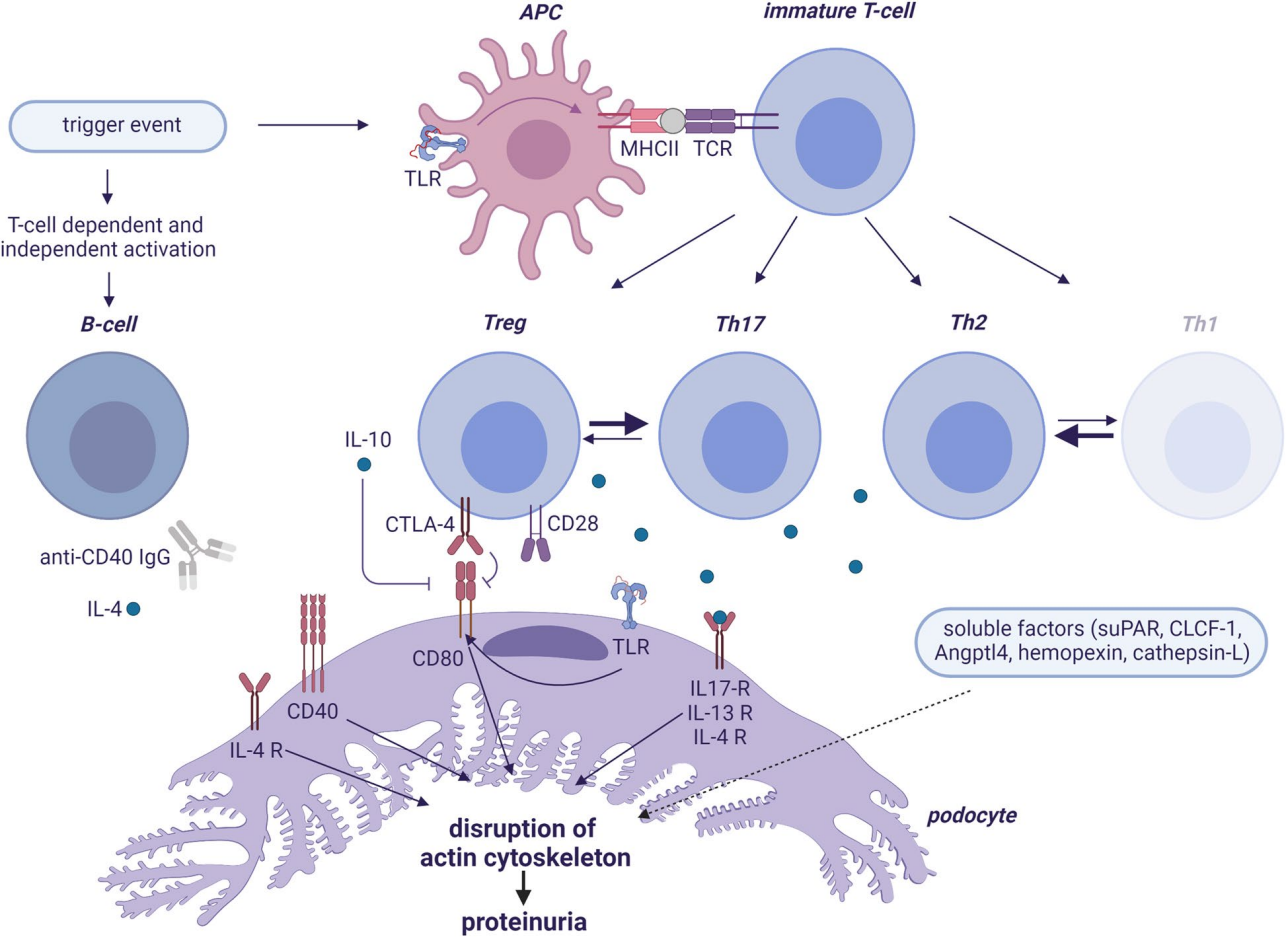
Schematic overview of the immunopathomechanism of idiopathic nephrotic syndrome; created by BioRender.com

Recent findings indicate that podocytes can act as antigen-presenting cells and be part of the adaptive immune system (Fig. 1). When exposed to stress conditions (infection, allergens, vaccination), podocytes have been shown to express receptors, characteristic for cells devoted to present antigens [80, 109–116]. For instance, exposure to low-dose LPS acting via TLR-4 was shown to upregulate CD80 in podocytes of wild-type and SCID mice, which in both cases caused nephrotic-range proteinuria, indicating that TLR-4 and CD80 in podocytes are possible effectors of proteinuria. LPS also induced CD80 expression in cultured podocytes with actin reorganization and morphological changes [106]. Furthermore, TLR-4 ligands induced CD80 expression in humans via an NF-kB-dependent pathway [111] and CD80 was co-localized with podocyte synaptopodin in human and murine kidney tissue specimens [106]. Finally, it has been shown that mice lacking CD80 are protected from LPS induced proteinuria, thus suggesting that this molecule is the mediator of LPS renal toxicity. Additionally, CD80 has its well-established receptor on T-cells, so that in immune-competent mice, it can also interact with CD28 on CD4+ T-cells, mediating their activation into Teff cells, and with CTLA-4 on Tregs, mediating the block of maturation towards a Teff phenotype, thus determining their activation (CD28) or inhibition (CTLA-4) [117–120]. Tregs may further inhibit the immune response by releasing soluble CTLA-4, IL-10, and transforming growth factor-beta (TGF-β) [121]. Human findings support this implication: the urinary CD80/CTLA-4 ratio was more than 100-fold higher in patients with relapse compared with those in remission [122]. Expression of glomerular CD80 was observed in renal biopsies of FSGS and treatment with Abatacept, a fusion CTLA-4 Ig molecule that inhibits CD80, can induce partial or complete remission in post-transplant recurrence of FSGS [123, 124]. CD80 expression can also be induced by polyinosinic-polycytidylic acid [111, 125], which stimulates TLR-3 and is structurally similar to the double-stranded RNA found in some viruses, which may explain the observation that MCD relapse is frequently triggered by an upper respiratory tract infection [126]. However, these data are not fully supported by others and the therapeutic potential of CD80 blockade is still a matter of debate [127].

Shimada et al. proposed the “two-hit” podocyte immune disorder underlying MCD [37]. The first hit is induction of podocyte expression of CD80 in response to a circulating factor such as cytokines, allergens, or microbial products. The second hit represents dysfunction of theauto-regulatory mechanisms (secretion of soluble CTLR-4, IL-10, and TGF-β by Tregs or downregulation of CD80 by podocytes itself), resulting in persistent CD80 expression and proteinuria.

Another co-stimulatory molecule in the adaptive immunity is CD40 [128, 129], which was observed to be constitutively expressed in human cultured podocytes and in glomerular biopsies of FSGS patients [80]. Circulating anti-CD40 IgG has also been additionally identified in the serum of patients with FSGS, but not in that of patients with other glomerular diseases [80]. In this study, purified anti-CD40 IgG from the sera of patients with FSGS was able to induce disruption of the podocyte actin cytoskeleton in vitro, and this effect was partially inhibited by blocking of CD40. Its ligand, CD40L may also exist in a soluble circulating formandthe CD40/CD40L complex mediates pro-inflammatory events [130], promotes redistribution of nephrin in podocytes and increases permeability to albumin in isolated glomeruli [131, 132].

### Experimental therapeutic options based on the immunological concept of INS

The resistance to the standard therapy (extensively reviewed by Trautmann et al. [133]) carries a high risk of progression to end-stage renal disease. Therefore, the translation of the promising findings gained in the basic research into the clinical practice is of high importance, in order to achieve advances in the treatment of nephrotic syndrome with higher effectiveness in reducing proteinuria [134].

Ofatumumab, a humanized anti-CD20 antibody, seems to be a new promising therapeutic approach. In comparison to RTX, ofatumumab has the advantage of higher affinity binding to B-cells and may be less prone to the development of antibodies against a murine fragment. Basu et al. administered ofatumumab to two patients with MCD and three patients with FSGS. All received multiple medications before this intervention, including two courses of RTX. After ofatumumab treatment, improvement of proteinuria and increase in the serum albumin levels were noted and no serious side effects were reported [135]. Currently, two randomized control trials are recruiting patients to test ofatumumab (*clinicaltrials.gov**NCT02394106 and* *clinicaltrials.gov**NCT02394119*) in therapy refractory INS. Ofatumumab has the potential to be an alternative for patients with native kidneys, which show either RTX resistance or RTX intolerance.

Another important candidate for the treatment of drug-resistant INS is abatacept, a fusion CTLA-4 Ig molecule that inhibits CD80, thus disrupting T-cell activation. Yu et al. treated five patients with FSGS, including four with recurrence after renal transplantation. They all achieved remission [123]. In line with that, one case report could confirm the positive effect of abatacept [136]. However, results from other studies on this molecule are discordant and do not confirm the original observation [137]. Therefore, abatacept may be a therapeutic option in CD80 positive cases, but further clinical trials are necessary.

One recent, additional therapy worth mentioning is lipid apheresis, which has been reported to be an effective measure to reduce proteinuria in patients with refractory nephrotic syndrome mostly in the Japanese population [138–140]. There are different mechanisms of action, which may contribute to its beneficial effect. Lipid apheresis on one hand reduces macrophage stimulation by lowering the oxidized LDL in the glomerulus, thus diminishing the production of inflammatory cytokines locally. On the other hand, it reduces Angptl4 levels by lowering the level of unbound FFAs in circulation, which in excess can cause proteinuria. Further non-immunological mechanisms of action are reviewed elsewhere [141, 142]. To note, a recent, excellent study, which attempts to distinguish between SSNS and SRNS [97] found that adiponectin and apolipoprotein A1, two proteins strongly related to lipid metabolism, are the ones of the strongest candidate biomarkers to predict steroid resistance, so that lipid apheresis could be considered particularly in patients with SRNS, but further clinical data is missing.

Mesenchymal stem cells (MSC) can serve as a backup option in multi-drug resistant INS via exerting paracrine action and thus causing a persistent reduction of several inflammatory molecules and circulating factors. Indeed, a pediatric patient with FSGS recurrence after kidney transplantation, resistant to plasmapheresis and RTX, presented a stable reduction of proteinuria after MSC infusion. No adverse events were recorded during or after infusion [143]. At the moment two open-label phase I clinical trials are recruiting patients to test the safety and efficacy of mesenchymal stromal cells *(**clinicaltrials.gov**NCT02382874*) and stem cells infusion (*clinicaltrials.govNCT02693366*) in multi-drug resistant FSGS.

Some of the molecules having a significant impact on proteinuria in animal models of INS did not fulfill the expectation in clinical settings such as anti-IL-2 antibodies [144], the anti-TGF-β antibody fresolimumab [145] or the anti-TNF-α antibody adalimumab [146]. Other biologicals proposed by basic science experiments are awaiting further investigations in nephrotic patients such as the anti-CD40 blocking antibody lucatumumab, anti-IL-13 antibodies or anti-IL-4 antibodies.

## Conclusion

In spite of the numerous experimental and clinical studies performed in the last decades, the immune pathogenesis of the non-genetic, idiopathic nephrotic syndrome is still not completely understood. It seems to be likely that INS is driven by a complex interplay between soluble factors, immunoregulatory cells, and podocytes that may vary between patients. Its outcome is determined by a multi-step control system, where defective regulatory steps may trigger and maintain foot process effacement and the persistence of proteinuria. Studies assessing patients’ individual profiles may help define precise targets for therapeutic intervention, leading to a more successful, personalized therapeutic approach.

## Data Availability

Not applicable

## Abbreviations

MCD: Minimal change disease
FSGS: Focal segmental glomerulosclerosis
INS: Idiopathic nephrotic syndrome
SSNS: Steroid-sensitive NS
SDNS: Steroid-dependent NS
SRNS: Steroid-resistant NS
IPEX: Immune dysregulation Polyendocrinopathy, Enteropathy, and X-linked syndrome
FoxP3: Forkhead box P3
Tregs: Regulatory T-cells
Teffs: Effector T-cells (Th17, Th1, Th2)
CTLA-4: Cytotoxic T-lymphocyte-associated protein 4
Bcl-2: B-cell lymphoma 2
c-mip: c-maf-inducing protein
RTX: Rituximab
suPAR: Soluble urokinase-type plasminogen activator receptor
CLCF-1: Cardiotrophin-like cytokine factor
Angptl4: Angiopoietin-like 4
LPS: Lipopolysaccharide
SCID: Severe combined immunodeficient
TLR: Toll-like receptor
NF-κB: Nuclear factor kappa-light-chain-enhancer of activated B cells
CD40L: CD40 ligand
GBM: Glomerular basal membrane
MSC: Mesenchymal stem cells
LDL: Low-density lipoprotein
FFA: Free fatty acid
